# mmCSM-PPI: predicting the effects of multiple point mutations on protein–protein interactions

**DOI:** 10.1093/nar/gkab273

**Published:** 2021-04-24

**Authors:** Carlos H M Rodrigues, Douglas E V Pires, David B Ascher

**Affiliations:** Computational Biology and Clinical Informatics, Baker Heart and Diabetes Institute, Melbourne, Victoria, Australia; Structural Biology and Bioinformatics, Department of Biochemistry and Pharmacology, University of Melbourne, Melbourne, Victoria, Australia; Systems and Computational Biology, Bio21 Institute, University of Melbourne, Melbourne, Victoria, Australia; Computational Biology and Clinical Informatics, Baker Heart and Diabetes Institute, Melbourne, Victoria, Australia; Structural Biology and Bioinformatics, Department of Biochemistry and Pharmacology, University of Melbourne, Melbourne, Victoria, Australia; Systems and Computational Biology, Bio21 Institute, University of Melbourne, Melbourne, Victoria, Australia; School of Computing and Information Systems, University of Melbourne, Melbourne, Victoria, Australia; Computational Biology and Clinical Informatics, Baker Heart and Diabetes Institute, Melbourne, Victoria, Australia; Structural Biology and Bioinformatics, Department of Biochemistry and Pharmacology, University of Melbourne, Melbourne, Victoria, Australia; Systems and Computational Biology, Bio21 Institute, University of Melbourne, Melbourne, Victoria, Australia; Department of Biochemistry, University of Cambridge, Cambridge, UK

## Abstract

Protein–protein interactions play a crucial role in all cellular functions and biological processes and mutations leading to their disruption are enriched in many diseases. While a number of computational methods to assess the effects of variants on protein–protein binding affinity have been proposed, they are in general limited to the analysis of single point mutations and have been shown to perform poorly on independent test sets. Here, we present mmCSM-PPI, a scalable and effective machine learning model for accurately assessing changes in protein–protein binding affinity caused by single and multiple missense mutations. We expanded our well-established graph-based signatures in order to capture physicochemical and geometrical properties of multiple wild-type residue environments and integrated them with substitution scores and dynamics terms from normal mode analysis. mmCSM-PPI was able to achieve a Pearson's correlation of up to 0.75 (RMSE = 1.64 kcal/mol) under 10-fold cross-validation and 0.70 (RMSE = 2.06 kcal/mol) on a non-redundant blind test, outperforming existing methods. Our method is freely available as a user-friendly and easy-to-use web server and API at http://biosig.unimelb.edu.au/mmcsm_ppi.

## Graphical Abstract

**Graphical Abstract:**
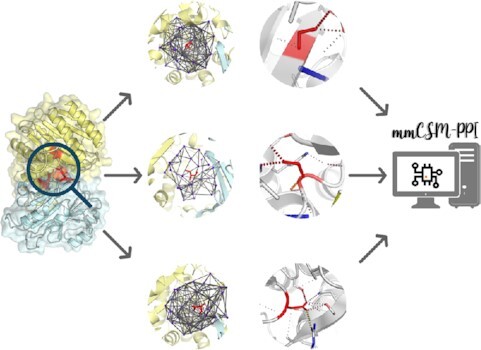
mmCSM-PPI: predicting the effects of multiple point mutations on protein–protein interactions.

## INTRODUCTION

Protein-protein interactions (PPIs) are a vital mechanism for regulation and coordination of most biological processes within the cell (1,2). Missense mutations are known to directly contribute to function disruption and are enriched at their interacting interface in many diseases (3–7). The ability to elucidate the underlying mechanisms by which point mutations affect PPI interactions is therefore essential for understanding how to modulate these interactions and the development of therapeutics to target them.

Significant efforts in the creation of manually curated databases compiling experimental data on the effects of mutations on protein stability and PPI binding affinity, most notably ThermomutDB (8), ProTherm (9), PROXiMATE (10) and SKEMPI (11,12), have greatly facilitated studies aiming to understand and predict how missense mutations affect PPIs. However, these have shown to perform poorly on independent test sets and are usually limited to predicting effects of single point mutations. Furthermore, to the best of our knowledge, little effort has been made towards accessibility of these methods to help integration into other analysis pipelines.

We have shown previously that representing protein structure as a graph is a powerful method for extracting structural signatures as distance patterns (13). These compile geometrical and physicochemical properties which can further be mined and applied in a broad range of areas, such as predicting the effects of single point missense mutations on protein stability (14–18), dynamics (16,17), interactions (15,19–25), genetic diseases (26–38) and drug resistance (39–53).

**Figure 1.**
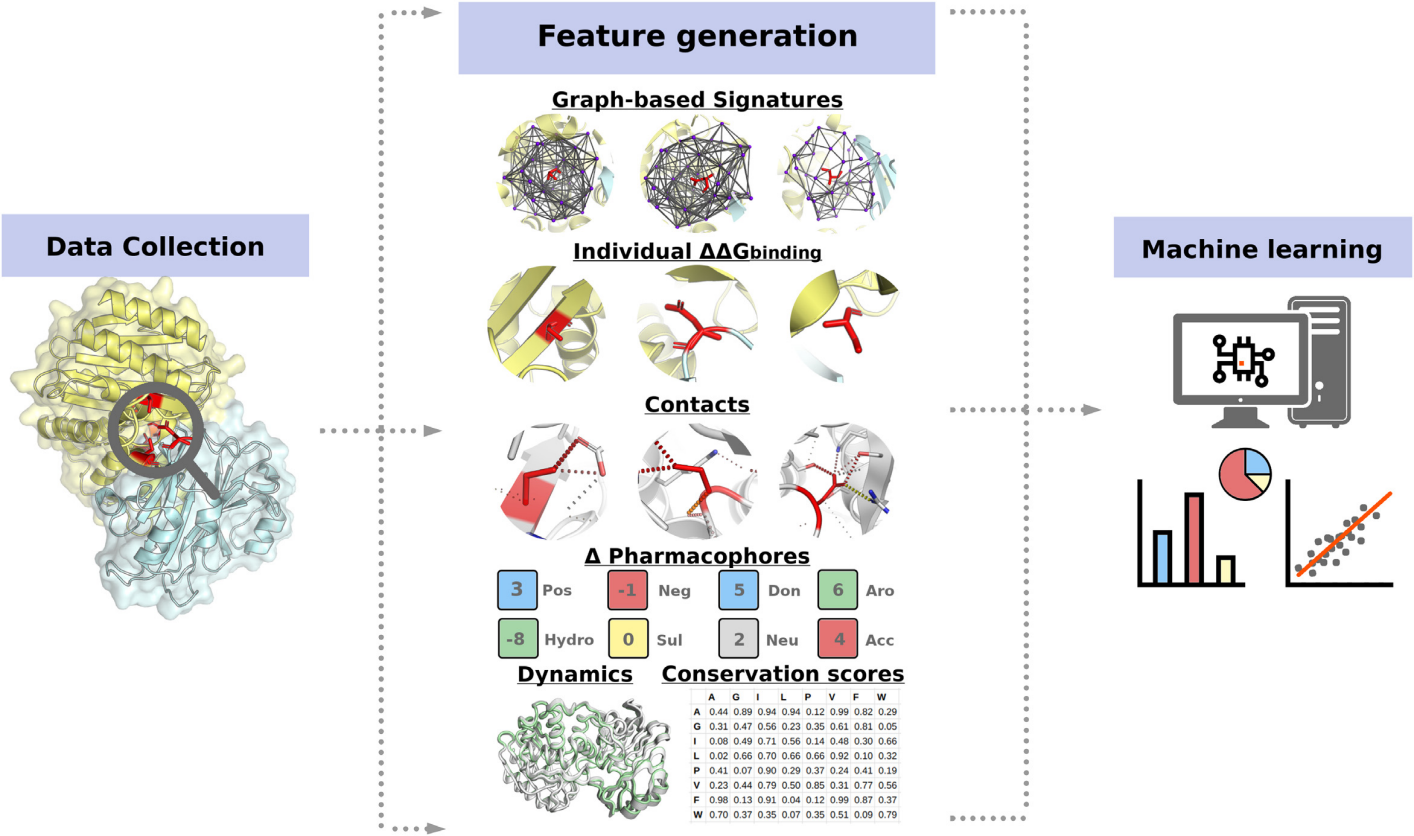
mmCSM-PPI methodology workflow. Experimental data on the effects of multiple missense mutations was collected from SKEMPI2 and mapped on their respective protein structures on the PDB. These were then used to generate physicochemical and geometrical properties in the form of graph-based signatures. In addition, six distinct types of complementary features were calculated to account for different mechanisms by which mutations may affect PPIs: (i) dynamic properties from NMA; (ii) wild-type residues environments; (iii) evolutionary and contact potential scores; (iv) non-covalent contacts; (v) wild-type inter-residue distances and (vi) the individual ΔΔG^binding^ for each point mutation. Feature selection was carried out with a stepwise greedy approach to avoid the curse of dimensionality and the best performing supervised learning algorithm was fine-tuned using the GridSearch function from the Scikit-learn Python library.

Here, we introduce mmCSM-PPI, a scalable and effective predictive model for assessing changes in PPI binding affinity caused by multiple missense mutations. We expanded our well-established graph-based signatures to allow for capturing physicochemical and geometrical properties of multiple wild-type residue environments, and integrated them with evolutionary scores, dynamics terms from Normal Mode Analysis (NMA) and non-covalent interactions for an accurate overall prediction (Figure 1).

## MATERIALS AND METHODS

### Datasets

The data used in this work was derived from SKEMPI2 (12), a manually curated database of experimental data on thermodynamics and kinetic parameters for wild-type and mutant protein–protein complexes which have been mapped to protein structures available on the Protein Data Bank (54). We were able to retrieve experimental information on 1721 multiple mutations, ranging from 2 to 27 point mutations, across 147 different protein–protein complexes (Supplementary Table S1). These had been primarily experimentally characterised by surface plasmon resonance and fluorescence methods (Supplementary Table S2 and Supplementary Figure S1).

Wild-type and mutant binding affinity parameters from SKEMPI2 were used to calculate the Gibbs free energy of binding as follows:

$$\begin{equation*}{{\Delta }}{{{G}}^{{{{\rm binding}}}}} = {\rm{ }}{{R}}{\rm{ }}{{T}}{\rm{ }}{\rm{ln}}\left( {{{{K}}_{\rm{D}}}} \right)\end{equation*}$$

where *R* = 1.9872 cal/K·mol is the ideal gas constant, *T* is the temperature (in Kelvin) and *K*_D_ is the affinity of the protein–protein complex.

The change in binding affinity upon mutation was calculated with the formulation previously described in SKEMPI2 and used in previous works:

$$\begin{equation*}{{\Delta \Delta }}{{{G}}^{{\rm{binding}}}} = {{\Delta }}{{{G}}^{{\rm{binding}}}}_{{\rm{WT}}} - {{\Delta }}{{{G}}^{{\rm{binding}}}}_{{\rm{MT}}}\end{equation*}$$

With positive values denoting mutations leading to an increased affinity and negative values denoting decreased binding affinity, given in kcal/mol. As shown in Supplementary Figure S2, the majority of entries in our dataset (1126) comprise double and triple mutants and for this work these were used as evidenced to train our predictive model. Furthermore, we explored the performance of our method on low-redundancy sets at complex and binding interface levels according to the definition used in SKEMPI2. The remaining 595 multiple point mutations (2 neutral, 153 increasing and 440 decreasing affinity), ranging from 4–27 mutations, were held out and used as a non-redundant blind test at mutation level for performance comparison.

The distribution of ΔΔ*G*^binding^ (Supplementary Figure S3A) depicts a clear bias towards mutations that decrease binding affinity (ΔΔ*G*^binding^ < 0 kcal/mol) in the training set. To minimize the imbalance nature of the dataset and how it would affect our predictive model, we also included modelled hypothetical reverse mutations in the training set (55,56). Unlike previous implementations, however, here we only modelled hypothetical reverse mutations for entries where –0.5 kcal/mol < ΔΔ*G*^binding^ < 0.5 kcal/mol to minimise uncertainties about the quality and biological implications of the modelled mutant structure (17). Therefore, the final training set used in this study includes 1344 entries, 12 neutral (ΔΔ*G*^binding^ = 0 kcal/mol), 347 increasing (ΔΔ*G*^binding^ < 0 kcal/mol) and 985 decreasing binding affinity (ΔΔ*G*^binding^ > 0 kcal/mol). All datasets used for training and test are freely available at http://biosig.unimelb.edu.au/mmcsm_ppi/data.

### Graph-based signatures

Our graph-based structural signatures framework is a well-established approach used to represent physicochemical and geometrical properties of protein structure and small molecules. In the past decade, our method has been widely used for assessing the effects of single point mutations on protein stability (14–16,18), PPI and antibody-antigen binding affinity (15,19,23,25), and small molecules toxicity (57–59). More recently, we have successfully expanded the applicability of our approach to investigate the impact of multiple point mutations on protein stability (17) and on antibody-antigen binding affinity (24).

In this work, for each point mutation, our signatures represent atoms of the wild-type residues as nodes and their interactions as edges, where their physicochemical properties are incorporated as labels according to amino acid residue properties (pharmacophores). The representation of each wild-type residue environment is then used to extract distance patterns between atoms characterised by their properties and compiled in signatures as cumulative distributions. Finally, the cumulative distributions are averaged based on the number of point mutations (Supplementary Figure S4).

### Modelling multiple mutation effects

Similarly to our previous implementation tackling the effects of single point mutations on PPI binding affinity (15,23), here we also incorporate complementary features to account for the different mechanisms by which multiple point mutations may affect PPIs. However, in this study, we calculated the sum and average values of each property in order to model the effects of multiple mutations. All features generated can be broadly classified into 6 different categories: (i) dynamics, obtained via normal mode analysis (60), (ii) residue environment properties (61), (iii) conservation, obtained by using scores from substitution tables (62), (iv) non-covalent contacts involving wild-type residues (63), (v) wild-type inter-residue distance and (vi) predicted ΔΔ*G*^binding^ for each single point mutation separately (23). A summary of features for each category is available in Supplementary Table S3.

### Machine learning

In this study we evaluated four distinct algorithms available on the scikit-learn Python library (64) on 10-fold cross-validation: Extra Trees, Random Forest, Gradient Boosting and XGBoost. The best performing algorithm used to build the final model was Extra Trees, based on different correlation coefficients (Pearson, Kendall and Spearman) and RMSE. Supplementary Table S4 summarises the performances of each algorithm. In order to avoid the curse of dimensionality and improve performance, we selected our features using an incremental stepwise greedy approach. Hyperparameter tuning was performed using the Gridsearch function also available on the scikit-learn library (Supplementary Table S5). Feature importance for the final predictive model is available on Supplementary Table S6. While two classes of features, graph-based signatures and individual mutation effects, were identified as contributing the most to the final model (as shown in Supplementary Table S7), their combination allowed for a significant increase in performance in the final model (*P*-value < 0.05), indicating they measure complementary aspects of mutation effects in PPIs.

## WEB SERVER

We have implemented mmCSM-PPI as a user-friendly and freely available web server (http://biosig.unimelb.edu.au/mmcsm_ppi). The server front end was developed using Materialize framework version 1.0.0, and the back end was built using Python via the Flask framework (version 1.0.2). The web server is hosted on a Linux Server running Apache2.

### Input

mmCSM-PPI can be used to either predict the effects of a list of mutations of interest or perform a systematic evaluation of all double and triple multiple mutations at a protein–protein interface (Supplementary Figure S5). In both cases, users are required to upload a file in PDB format or provide a valid PDB accession code with the structure of a protein–protein complex. For user-specified variants, mutations can be provided using a text field or uploaded as a plain text file with one multiple mutation per line. Each entry must be separated by a semicolon (;) and each point mutation must be represented as the chain identifier, blank space, the one-letter code for the wild-type, residue position and the one-letter code for the mutant. For the systematic evaluation option, users must provide a chain identifier from which interfaces will be automatically identified and all possible permutations of double and triple mutations assessed. Examples and format descriptions are available in both submission page and help page via the top navigation menu.

An Application Programming Interface (API) to assist users in integrating our predictive tool into their research pipelines is also available. Input fields follow the same format previously described for our web server implementation. All jobs submitted are labelled with a unique identifier which is used to query the status of the job. A full description of the API, including examples using curl and Python are available at http://biosig.unimelb.edu.au/mmcsm_ppi/api.

### Output

For both types of submissions, manual input and systematic evaluation, mmCSM-PPI outputs the predictions for all entries as a downloadable table where the predicted effects of multiple mutations on ΔΔ*G*^binding^ is given in kcal/mol. For the systematic evaluation option, the server shows the top 100 increasing/decreasing affinity entries. Additionally, individual predictions for each point mutation are available, generated using mCSM-PPI2 (23), and are shown alongside the average distance among the wild-type residues. Finally, an interactive 3D viewer, built using the NGL viewer (65), allows for the analysis of non-covalent interactions involving wild-type residues for each point mutation, calculated using Arpeggio (63), for a particular entry. Users can alternate the residues and interactions being displayed by selecting different entries from the table (Supplementary Figure S6).

## VALIDATION

### Performance on cross-validation

**Figure 2.**
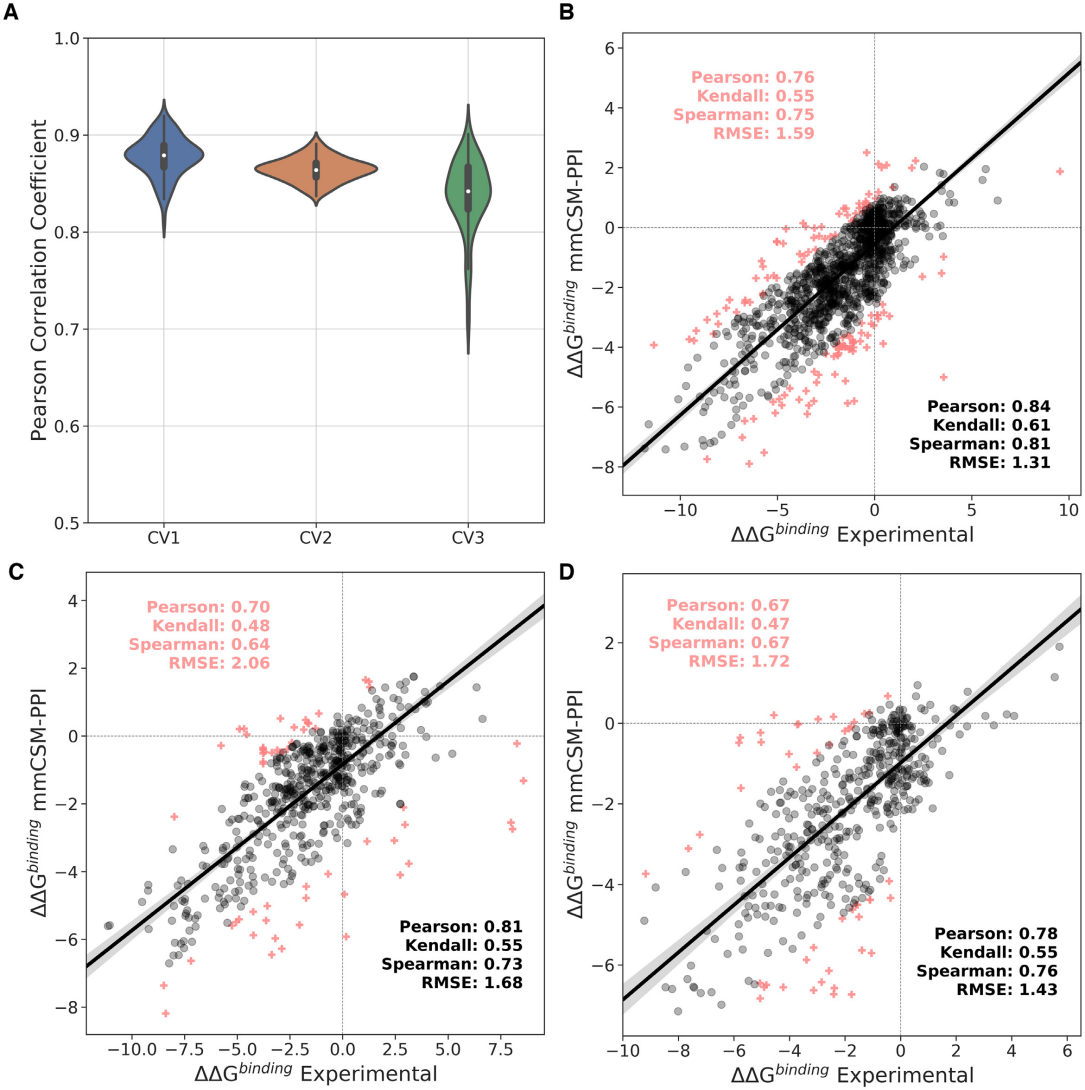
mmCSM-PPI performance on cross-validation and non-redundant blind-tests. (**A**) The performance of mmCSM-PPI on bootstrapped 5-fold cross validation (CV1), using 50% of the data as a blind test (CV2) and limiting the number of mutations per complex (CV3). The robustness of mmCSM-PPI was further assessed using low redundancy at the (**B**) complex level, (**C**) using all data with three or more mutations as a blind test, and (**D**) at the mutation level. Outliers are shown as red crosses.

We evaluated the performance of mmCSM-PPI across 5 different types of cross-validations on our training set. First, we randomly selected 80% of the data for training and remaining 20% for testing, repeated 100 times (CV1). Our method achieved Pearson's, Kendall's and Spearman's correlations of 0.87, 0.68 and 0.85 respectively, with small deviations across repetitions (σ = 0.02), and average RMSE of 1.41 kcal/mol (σ = 0.21). Using an analogous setup, but varying the proportion of data split for train and test (50% each set) (CV2), the performance was consistent with the previous experiment, and the predictive model achieved a Pearson's, Kendall's and Spearman's correlations of 0.86, 0.66 and 0.84 (σ = 0.01 for all coefficients), respectively (Figure 2A), and RMSE = 1.55 kcal/mol (σ = 0.14).

Since the entries in our dataset were not uniformly distributed across all protein–protein complexes (Supplementary Table S8), we evaluated the performance of our approach by randomly sampling up to 10 mutations per protein complex, repeated 10 times (generating 10 subsets), followed by randomly selecting 80% of entries for training and remaining 20% for testing, also repeated 10 times (CV3). For this type of cross-validation, our predictive model was able to achieve Pearson's, Kendall's and Spearman's correlations of 0.83, 0.63 and 0.81, again with small deviations over the repetitions (σ = 0.03) (Figure 2A), and average RMSE = 1.85 kcal/mol (σ = 0.40).

Finally, we assessed the robustness of mmCSM-PPI on low-redundancy sets at complex (CV4) and interface (CV5) levels. The former was implemented using leave-one-complex-out cross-validation, where all mutations for a particular complex were retained for test and the remaining for training the predictive model. Overall, our predictive model achieved Pearson's, Kendall's and Spearman's correlations of 0.76, 0.55 and 0.75 respectively, and RMSE of 1.59 kcal/mol (Figure 2B). On leave-one-binding-site-out (CV5), where all mutations for protein–protein complexes sharing similar binding sites, according to data on SKEMPI2, were used for testing and the remaining for training, our method was able to achieve Pearson's, Kendall's and Spearman's correlations of 0.73, 0.54 and 0.74, respectively (RMSE = 1.40 kcal/mol).

### Blind test

While mmCSM-PPI was trained using a subset containing only double and triple mutants, the performance of our final model was further evaluated using a non-redundant blind set at the mutation level of experimentally measured effects of 595 constructs with at least four point mutations, also derived from SKEMPI2. Across this dataset, mmCSM-PPI achieved Pearson's, Kendall's and Spearman's correlation coefficients of 0.70, 0.48 and 0.64, respectively, and RMSE of 2.02 kcal/mol, significantly outperforming FoldX (66) and Discovery Studio (*P*-value < 0.05, Table 1). After removing 10% of outliers, the performance of our predictive model increased to 0.81, 0.55 and 0.73 for Pearson's, Kendall's and Spearman's correlations, respectively, and RMSE of 1.68 kcal/mol (Figure 2C). The majority of outliers (∼70%) comprise mutations with extreme effects to PPI binding affinity (4 kcal/mol < |ΔΔ*G*^binding^| < 11 kcal/mol) and entries with 10 or more point mutations. Reassuringly, however, our final model demonstrated balanced predictive performance across both stabilising and destabilising mutations, achieving an overall accuracy of 87% and precisions of 74% and 89% on mutations that increase and decrease binding affinity, respectively.

**Table 1.** Performance comparison of mmCSM-PPI2 on a non-redundant blind test comprising entries with four or more mutations

Method	Pearson	Kendall	Spearman	RMSE (kcal/mol)	MCC	AUC
**mmCSM-PPI**	**0.70**	**0.48**	**0.64**	**2.02**	**0.53**	**0.72**
Discovery Studio	0.39*	0.29^#^	0.41^+^	3.07^a^	0.30	0.66
FoldX	0.39*	0.25^#^	0.37^+^	5.27^a^	0.22	0.61^b^

*P-value < 0.05 by Fisher r-to-z transformation test.

^#^ *P* < 0.05 by transforming tau-to-r followed by Fisher r-to-z transformation.

^+^ *P* < 0.05 by transforming rho-to-r followed by Fisher r-to-z transformation.

^a^ *P* < 0.05 by Diebold–Mariano test.

^b^ *P* < 0.05 by *t*-test.

Given the inherent imbalance between increasing and decreasing affinity mutations in the dataset, we further assessed the performance of our method on these respective classes separately. On mutations that decrease binding affinity, mmCSM-PPI achieves Pearson's, Kendall's and Spearman's correlations of 0.72, 0.46 and 0.64 respectively, with an RMSE = 1.67 kcal/mol, outperforming FoldX and Discovery Studio. For mutations that increase binding affinity all three methods show similar performance (Supplementary Table S9). Finally, we tested the ability to use the predicted ΔΔ*G*^binding^ values from mmCSM-PPI to differentiate between mutations that increase from those that decrease binding affinity (Supplementary Table S10). Overall, our method has proven to be the most robust when compared with FoldX and Discovery Studio, achieving an AUC and MCC of 0.72 and 0.53, respectively, when evaluated on mutations where |ΔΔ*G*^binding^| < 1 kcal/mol.

We further evaluated the generalisation capabilities of our model on another independent test set, non-redundant at the mutation level. Four hundred and ninety multiple point mutations were randomly selected across 81 different PPI as a blind test, with the remaining being used for training purposes. Across the non-redundant blind test, mmCSM-PPI achieved Pearson's, Kendall's and Spearman's correlations of 0.67, 0.47 and 0.67, respectively (RMSE = 1.72 kcal/mol), performance consistent with previous independent tests, highlighting robustness of the method (Figure 2D).

The performance of mmCSM-PPI was compared to Discovery Studio and FoldX (Supplementary Table S11), which demonstrated that our approach significantly outperformed both in all metric evaluations (Supplementary Table S11). We also compared the performance of our method with ZEMu (67), a tool that uses a dynamical equilibration under a physics-based force field for a limited residue environment, followed by binding affinity evaluation with FoldX. In this case since ZEMu has only reported predictions for multiple mutations on the first version of SKEMPI, here we trained a predictive model with all double and triple mutants except for those available on the first release of SKEMPI. Therefore, the dataset used to compare the two methods comprises 272 entries (1 neutral, 52 increasing and 219 decreasing binding affinity) across 24 protein–protein complexes, ranging from 2 to 15 point mutations. mmCSM-PPI achieved Pearson's, Kendall's and Spearman's correlations of 0.73, 0.56 and 0.75 (RMSE = 1.72 kcal/mol), respectively, significantly higher (*P*-value < 0.05) than ZEMu (Pearson's, Kendall's and Spearman's correlations of 0.64, 0.46 and 0.65, respectively, and RMSE = 2.11 kcal/mol). On 90% of the dataset, our method achieves up to 0.83, 0.65 and 0.84 on Pearson's, Kendall's and Spearman's, respectively (RMSE = 1.49 kcal/mol).

## CONCLUSION

Here, we present mmCSM-PPI, a web server that integrates our well-established graph-based signatures framework with evolutionary scores, dynamics properties and non-covalent interactions for accurately predicting changes in PPI binding affinity caused by multiple point mutations. Our method has shown to be robust when evaluated across different types of cross-validations and outperformed existing tools in a non-redundant blind test set. We anticipate mmCSM-PPI to be of great value for the study of how multiple mutations affect PPI binding affinity and to a variety of applications, ranging from protein functional analysis, optimisation of binding affinity and understanding the role of mutations in diseases. In addition, mmCSM-PPI includes an API to assist users when integrating our predictions into their research pipelines. Our method is freely available as a user-friendly and easy-to-use web server at http://biosig.unimelb.edu.au/mmcsm_ppi.

## DATA AVAILABILITY

mmCSM-PPI predictive models are freely available either as a user-friendly web interface and as an API for programmatic access at http://biosig.unimelb.edu.au/mmcsm_ppi. No login or license is required. All data sets used to train and validate predicted models are publicly available for download at http://biosig.unimelb.edu.au/mmcsm_ppi/data.

## Supplementary Material

gkab273_Supplemental_File
