# Supplementary material for: mmCSM-PPI: predicting the effects of multiple point mutations on protein–protein interactions
Source: Nucleic Acids Res. 2021 Apr 24;49(W1):W417–24. doi: 10.1093/nar/gkab273 (PMC8262703; doi:10.1093/nar/gkab273)
Supplement: gkab273_Supplemental_File [file gkab273_supplemental_file.docx]

SUPPLEMENTARY MATERIAL

mmCSM-PPI: predicting the effects of multiple point mutations on protein-protein interactions

Carlos H. M. Rodrigues ^1,2,3^, Douglas E. V. Pires ^1,2,3,4,*^, David B. Ascher ^1,2,3,5,*^

^1^ Computational Biology and Clinical Informatics, Baker Heart and Diabetes Institute, Melbourne, Victoria

^2^ Structural Biology and Bioinformatics, Department of Biochemistry and Molecular Biology, University of Melbourne, Melbourne, Victoria

^3^ Systems and Computational Biology, Bio21 Institute, University of Melbourne, Melbourne, Victoria

^4^ School of Computing and Information Systems, University of Melbourne, Melbourne, Victoria

^5^ Department of Biochemistry, University of Cambridge, Cambridge, UK

^*^To whom correspondence should be addressed D.B.A. Tel: +61 90354794; Email: [david.ascher@unimelb.edu.au](mailto:david.ascher@unimelb.edu.au). Correspondence may also be addressed to D.E.V.P. [douglas.pires@unimelb.edu.au](mailto:douglas.pires@unimelb.edu.au).

# Extracting Binding Free Energy values from FoldX and Discovery Studio

In order to extract the effects of mutations on binding free energy from FoldX, we first generated mutant structures using the command BuildModel and then generated ΔGBinding for wild-type and mutant structures using the function AnalyseComplex. Information on the groups of molecules (chains) participating in the interaction and the change in binding free energy was then calculated using the definitions available in SKEMPI2:

ΔΔG^binding^ = ΔG^binding^_WT_ - ΔG^binding^_MT_

For Discovery Studio, we obtained values for changes in binding free energy using the Pipeline Plot 2018 module, running on CHARMM force-field and default configurations.

# TABLES

**Table S1** - Description of PPI structures present in the dataset used to build mmCSM-PPI. The majority of structures have been generated using X-ray crystallography and a minor fraction using Nuclear Magnetic Resonance (NMR). For the latter, resolution values have been set as “NA”.

| PDB ID | Description | Experiment | Resolution |
| --- | --- | --- | --- |
| 1cz8 | Vascular endothelial growth factor in complex with an affinity matured antibody | x-ray diffraction | 2.40 |
| 5m2o | R. flavefaciens' third scab cohesin in complex with a group 1 dockerin | x-ray diffraction | 1.26 |
| 4l3e | The complex between high affinity tcr dmf5(alpha-d26y,beta-l98w) and human class i mhc hla-a2 with the bound mart-1(26-35)(a27l) peptide | x-ray diffraction | 2.56 |
| 1mhp | Crystal structure of a chimeric alpha1 integrin i-domain in complex with the fab fragment of a humanized neutralizing antibody | x-ray diffraction | 2.80 |
| 2c5d | Structure of a minimal gas6-axl complex | x-ray diffraction | 3.30 |
| 1yqv | The crystal structure of the antibody fab hyhel5 complex with lysozyme at 1.7a resolution | x-ray diffraction | 1.70 |
| 3idx | Crystal structure of hiv-gp120 core in complex with cd4-binding site antibody b13, space group c222 | x-ray diffraction | 2.50 |
| 2oob | Crystal structure of the uba domain from cbl-b ubiquitin ligase in complex with ubiquitin | x-ray diffraction | 1.90 |
| 4gnk | Crystal structure of galphaq in complex with full-length human plcbeta3 | x-ray diffraction | 4.00 |
| 1qab | The structure of human retinol binding protein with its carrier protein transthyretin reveals interaction with the carboxy terminus of rbp | x-ray diffraction | 3.20 |
| 1c1y | Crystal structure of rap.gmppnp in complex with the ras- binding-domain of c-raf1 kinase (rafrbd). | x-ray diffraction | 1.90 |
| 4b0m | Complex of the caf1an usher domain, caf1m chaperone and caf1 subunit from yersinia pestis | x-ray diffraction | 1.80 |
| 4g2v | Structure complex of lgn binding with frmpd1 | x-ray diffraction | 2.40 |
| 1tm1 | Crystal structure of the complex of subtilisin bpn' with chymotrypsin inhibitor 2 | x-ray diffraction | 1.70 |
| 3hg1 | Germline-governed recognition of a cancer epitope by an immunodominant human t cell receptor | x-ray diffraction | 3.00 |
| 1dqj | Crystal structure of the anti-lysozyme antibody hyhel-63 complexed with hen egg white lysozyme | x-ray diffraction | 2.00 |
| 1n8z | Crystal structure of extracellular domain of human her2 complexed with herceptin fab | x-ray diffraction | 2.52 |
| 1ycs | P53-53bp2 complex | x-ray diffraction | 2.20 |
| 1y4a | Crystal structure of the complex of subtilisin bpn' with chymotrypsin inhibitor 2 m59r/e60s mutant | x-ray diffraction | 1.60 |
| 1dan | Complex of active site inhibited human blood coagulation factor viia with human recombinant soluble tissue factor | x-ray diffraction | 2.00 |
| 1yy9 | Structure of the extracellular domain of the epidermal growth factor receptor in complex with the fab fragment of cetuximab/erbitux/imc- c225 | x-ray diffraction | 2.61 |
| 4jfd | Preservation of peptide specificity during tcr-mhc contact dominated affinity enhancement of a melanoma-specific tcr | x-ray diffraction | 2.46 |
| 4jfe | Preservation of peptide specificity during tcr-mhc contact dominated affinity enhancement of a melanoma-specific tcr | x-ray diffraction | 2.70 |
| 1lfd | Crystal structure of the active ras protein complexed with the ras- interacting domain of ralgds | x-ray diffraction | 2.10 |
| 4ra0 | An engineered axl 'decoy receptor' effectively silences the gas6-axl signaling axis | x-ray diffraction | 3.07 |
| 3uih | Crystal structure of human survivin in complex with smac/diablo(1-15) peptide | x-ray diffraction | 2.40 |
| 2pye | Crystal structures of high affinity human t-cell receptors bound to pmhc revealnative diagonal binding geometry tcr clone c5c1 complexed with mhc | x-ray diffraction | 2.30 |
| 3bdy | Dual specific bh1 fab in complex with vegf | x-ray diffraction | 2.60 |
| 1ak4 | Human cyclophilin a bound to the amino-terminal domain of hiv-1 capsid | x-ray diffraction | 2.36 |
| 1gc1 | Hiv-1 gp120 core complexed with cd4 and a neutralizing human antibody | x-ray diffraction | 2.50 |
| 3be1 | Dual specific bh1 fab in complex with the extracellular domain of her2/erbb-2 | x-ray diffraction | 2.90 |
| 4mnq | Tcr-peptide specificity overrides affinity enhancing tcr-mhc interactions | x-ray diffraction | 2.74 |
| 1E50 | Aml1/cbfbeta complex | x-ray diffraction | 2.60 |
| 2dvw | Structure of the oncoprotein gankyrin in complex with s6 atpase of the 26s proteasome | x-ray diffraction | 2.30 |
| 3qib | Crystal structure of the 2b4 tcr in complex with mcc/i-ek | x-ray diffraction | 2.70 |
| 1a4y | Ribonuclease inhibitor-angiogenin complex | x-ray diffraction | 2.00 |
| 1mq8 | Crystal structure of alphal i domain in complex with icam-1 | x-ray diffraction | 3.30 |
| 1gua | Human rap1a, residues 1-167, double mutant (e30d,k31e) complexed with gppnhp and the ras-binding-domain of human c-raf1, residues 51-131 | x-ray diffraction | 2.00 |
| 1rew | Structural refinement of the complex of bone morphogenetic protein 2 and its type ia receptor | x-ray diffraction | 1.86 |
| 4jeu | Crystal structure of munc18a and syntaxin1 with native n-terminus complex | x-ray diffraction | 3.20 |
| 1efn | Hiv-1 nef protein in complex with r96i mutant fyn sh3 domain | x-ray diffraction | 2.50 |
| 2o3b | Crystal structure complex of nuclease a (nuca) with intra-cellular inhibitor nuia | x-ray diffraction | 2.30 |
| 1kne | Chromo domain of hp1 complexed with histone h3 tail containing trimethyllysine 9 | x-ray diffraction | 2.40 |
| 2b2x | Vla1 rdeltah i-domain complexed with a quadruple mutant of the aqc2 fab | x-ray diffraction | 2.20 |
| 4uyq | High resolution structure of the third cohesin scac in complex with the scab dockerin with a mutation in the c-terminal helix (in to si) from acetivibrio cellulolyticus displaying a type i interaction. | x-ray diffraction | 1.81 |
| 4ftv | The complex between the high affinity version of a6 tcr (a6c134) and human class i mhc hla-a2 with the bound tax nonameric peptide | x-ray diffraction | 2.74 |
| 1mah | Fasciculin2-mouse acetylcholinesterase complex | x-ray diffraction | 3.20 |
| 3d3v | The complex between tcr a6 and human class i mhc hla-a2 with the modified htlv-1 tax (y5(3,4-difluorophenylalanine)) peptide | x-ray diffraction | 2.80 |
| 4ofy | Crystal structure of the complex of syg-1 d1-d2 and syg-2 d1-d4 | x-ray diffraction | 3.30 |
| 3l5x | Crystal structure of the complex between il-13 and h2l6 fab | x-ray diffraction | 1.90 |
| 1k8r | Crystal structure of ras-bry2rbd complex | x-ray diffraction | 3.00 |
| 3u82 | Binding of herpes simplex virus glycoprotein d to nectin-1 exploits host cell adhesion | x-ray diffraction | 3.16 |
| 5cyk | Structure of ytm1 bound to the c-terminal domain of erb1-r486e | x-ray diffraction | 3.00 |
| 3lzf | Crystal structure of fab 2d1 in complex with the 1918 influenza virus hemagglutinin | x-ray diffraction | 2.80 |
| 3vr6 | Crystal structure of amp-pnp bound enterococcus hirae v1-atpase [bv1] | x-ray diffraction | 2.68 |
| 1bd2 | Complex between human t-cell receptor b7, viral peptide (tax) and mhc class i molecule hla-a 0201 | x-ray diffraction | 2.50 |
| 2pcc | Crystal structure of a complex between electron transfer partners, cytochrome c peroxidase and cytochrome c | x-ray diffraction | 2.30 |
| 1gl0 | Structure of the complex between bovine alpha-chymotrypsin and pmp-d2v, an inhibitor from the insect locusta migratoria | x-ray diffraction | 3.00 |
| 1gl1 | Structure of the complex between bovine alpha-chymotrypsin and pmp-c, an inhibitor from the insect locusta migratoria | x-ray diffraction | 2.10 |
| 3mzg | Crystal structure of a human prolactin receptor antagonist in complex with the extracellular domain of the human prolactin receptor | x-ray diffraction | 2.10 |
| 5xco | Crystal structure of human k-ras g12d mutant in complex with gdp and cyclic inhibitory peptide | x-ray diffraction | 1.25 |
| 4gxu | Crystal structure of antibody 1f1 bound to the 1918 influenza hemagglutinin | x-ray diffraction | 3.29 |
| 3sgb | Structure of the complex of streptomyces griseus protease b and the third domain of the turkey ovomucoid inhibitor at 1.8 angstroms resolution | x-ray diffraction | 1.80 |
| 3s9d | Binary complex between ifna2 and ifnar2 | x-ray diffraction | 2.00 |
| 1vfb | Bound water molecules and conformational stabilization help mediate an antigen-antibody association | x-ray diffraction | 1.80 |
| 1jtg | Crystal structure of tem-1 beta-lactamase / beta-lactamase inhibitor protein complex | x-ray diffraction | 1.73 |
| 1he8 | Ras g12v - pi 3-kinase gamma complex | x-ray diffraction | 3.00 |
| 4k71 | Crystal structure of a high affinity human serum albumin variant bound to the neonatal fc receptor | x-ray diffraction | 2.40 |
| 3kud | Complex of ras-gdp with rafrbd(a85k) | x-ray diffraction | 2.15 |
| 1xxm | The modular architecture of protein-protein binding site | x-ray diffraction | 1.90 |
| 2nyy | Crystal structure of botulinum neurotoxin type a complexed with monoclonal antibody cr1 | x-ray diffraction | 2.61 |
| 1r0r | 1.1 angstrom resolution structure of the complex between the protein inhibitor, omtky3, and the serine protease, subtilisin carlsberg | x-ray diffraction | 1.10 |
| 5tar | Crystal structure of farnesylated and methylated kras4b in complex with pde-delta (crystal form ii - with ordered hypervariable region) | x-ray diffraction | 1.90 |
| 3se4 | Human ifnw-ifnar ternary complex | x-ray diffraction | 3.50 |
| 3se3 | Human ifna2-ifnar ternary complex | x-ray diffraction | 4.00 |
| 2kso | Epha2:ship2 sam:sam complex | NMR | NA |
| 1ppf | X-ray crystal structure of the complex of human leukocyte elastase (pmn elastase) and the third domain of the turkey ovomucoid inhibitor | x-ray diffraction | 1.80 |
| 4krl | Nanobody/vhh domain 7d12 in complex with domain iii of the extracellular region of egfr, ph 6.0 | x-ray diffraction | 2.85 |
| 4kro | Nanobody/vhh domain ega1 in complex with the extracellular region of egfr | x-ray diffraction | 3.05 |
| 3eg5 | Crystal structure of mdia1-tsh gbd-fh3 in complex with cdc42-gmppnp | x-ray diffraction | 2.70 |
| 2p5e | Crystal structures of high affinity human t-cell receptors bound to pmhc reveal native diagonal binding geometry | x-ray diffraction | 1.89 |
| 1ahw | A complex of extracellular domain of tissue factor with an inhibitory fab (5g9) | x-ray diffraction | 3.00 |
| 2ny7 | Hiv-1 gp120 envelope glycoprotein complexed with the broadly neutralizing cd4-binding-site antibody b12 | x-ray diffraction | 2.30 |
| 2g2u | Crystal structure of the shv-1 beta-lactamase/beta-lactamase inhibitor protein (blip) complex | x-ray diffraction | 1.60 |
| 1fcc | Crystal structure of the c2 fragment of streptococcal protein g in complex with the fc domain of human igg | x-ray diffraction | 3.20 |
| 1brs | Protein-protein recognition: crystal structural analysis of a barnase- barstar complex at 2.0-a resolution | x-ray diffraction | 2.00 |
| 1a22 | Human growth hormone bound to single receptor | x-ray diffraction | 2.60 |
| 2vn5 | The clostridium cellulolyticum dockerin displays a dual binding mode for its cohesin partner | x-ray diffraction | 1.90 |
| 1bj1 | Vascular endothelial growth factor in complex with a neutralizing antibody | x-ray diffraction | 2.40 |
| 3ngb | Crystal structure of broadly and potently neutralizing antibody vrc01 in complex with hiv-1 gp120 | x-ray diffraction | 2.68 |
| 5e9d | Rd-1 mart-1 high bound to mart-1 decameric peptide (ela) in complex with hla-a2 | x-ray diffraction | 2.51 |
| 4yh7 | Crystal structure of ptpdelta ectodomain in complex with il1rapl1 | x-ray diffraction | 4.40 |
| 1jrh | Complex (antibody/antigen) | x-ray diffraction | 2.80 |
| 1c4z | Structure of an e6ap-ubch7 complex: insights into the ubiquitination pathway | x-ray diffraction | 2.60 |
| 4uyp | High resolution structure of the third cohesin scac in complex with the scab dockerin with a mutation in the n-terminal helix (in to si) from acetivibrio cellulolyticus displaying a type i interaction. | x-ray diffraction | 1.49 |
| 1fss | Acetylcholinesterase (e.c. 3.1.1.7) complexed with fasciculin-ii | x-ray diffraction | 3.00 |
| 5c6t | Crystal structure of hcmv glycoprotein b in complex with 1g2 fab | x-ray diffraction | 3.60 |
| 4gu0 | Crystal structure of lsd2 with h3 | x-ray diffraction | 3.10 |
| 3uii | Crystal structure of human survivin in complex with h3(1-10) peptide | x-ray diffraction | 2.60 |
| 3aaa | Crystal structure of actin capping protein in complex with v-1 | x-ray diffraction | 2.20 |
| 1b41 | Human acetylcholinesterase complexed with fasciculin-ii, glycosylated protein | x-ray diffraction | 2.76 |
| 4nkq | Structure of a cytokine receptor complex | x-ray diffraction | 3.30 |
| 1cho | Crystal and molecular structures of the complex of alpha-*chymotrypsin with its inhibitor turkey ovomucoid third domain at 1.8 angstroms resolution | x-ray diffraction | 1.80 |
| 2wpt | The crystal structure of im2 in complex with colicin e9 dnase | x-ray diffraction | 1.78 |
| 2nz9 | Crystal structure of botulinum neurotoxin type a complexed with monoclonal antibody ar2 | x-ray diffraction | 3.79 |
| 4wnd | Crystal structure of the tpr domain of lgn in complex with frmpd4/preso1 at 1.5 angstrom resolution | x-ray diffraction | 1.50 |
| 3pwp | The complex between tcr a6 and human class i mhc hla-a2 with the bound hud peptide | x-ray diffraction | 2.69 |
| 4j2l | Crystal structure of axh domain complexed with capicua | x-ray diffraction | 3.15 |
| 1b2u | Structural response to mutation at a protein-protein interface | x-ray diffraction | 2.10 |
| 1b2s | Structural response to mutation at a protein-protein interface | x-ray diffraction | 1.82 |
| 1ao7 | Complex between human t-cell receptor, viral peptide (tax), and hla-a 0201 | x-ray diffraction | 2.60 |
| 1dvf | Idiotopic antibody d1.3 fv fragment-antiidiotopic antibody e5.2 fv fragment complex | x-ray diffraction | 1.90 |
| 2ccl | The s45a, t46a mutant of the type i cohesin-dockerin complex from the cellulosome of clostridium thermocellum | x-ray diffraction | 2.03 |
| 1bp3 | The xray structure of a growth hormone-prolactin receptor complex | x-ray diffraction | 2.90 |
| 2qj9 | Crystal structure analysis of bmp-2 in complex with bmpr-ia variant b1 | x-ray diffraction | 2.44 |
| 2noj | Crystal structure of ehp / c3d complex | x-ray diffraction | 2.70 |
| 5ufe | Wild-type k-ras(gnp)/r11.1.6 complex | x-ray diffraction | 2.30 |
| 1kbh | Mutual synergistic folding in the interaction between nuclear receptor coactivators cbp and actr | NMR | NA |
| 3hfm | Structure of an antibody-antigen complex. crystal structure of the hy/hel-10 fab-lysozyme complex | x-ray diffraction | 3.00 |
| 3qdg | The complex between tcr dmf5 and human class i mhc hla-a2 with the bound mart-1(26-35)(a27l) peptide | x-ray diffraction | 2.69 |
| 3qdj | The complex between tcr dmf5 and human class i mhc hla-a2 with the bound mart-1(27-35) nonameric peptide | x-ray diffraction | 2.30 |
| 4yfd | Crystal structure ptp delta ig1-fn2 in complex with il-1racp | x-ray diffraction | 3.25 |
| 2j0t | Crystal structure of the catalytic domain of mmp-1 in complex with the inhibitory domain of timp-1 | x-ray diffraction | 2.54 |
| 5ufq | K-rasg12d(gnp)/r11.1.6 complex | x-ray diffraction | 2.20 |
| 3mzw | Her2 extracelluar region with affinity matured 3-helix affibody zher2:342 | x-ray diffraction | 2.90 |
| 3g6d | Crystal structure of the complex between cnto607 fab and il-13 | x-ray diffraction | 3.20 |
| 4x4m | Structure of fcgammari in complex with fc reveals the importance of glycan recognition for high affinity igg binding | x-ray diffraction | 3.49 |
| 3m63 | Crystal structure of ufd2 in complex with the ubiquitin-like (ubl) domain of dsk2 | x-ray diffraction | 2.40 |
| 4myw | Structure of hsv-2 gd bound to nectin-1 | x-ray diffraction | 3.19 |
| 4e6k | 2.0 a resolution structure of pseudomonas aeruginosa bacterioferritin (bfrb) in complex with bacterioferritin associated ferredoxin (bfd) | x-ray diffraction | 2.00 |
| 1b3s | Structural response to mutation at a protein-protein interface | x-ray diffraction | 2.39 |
| 1z7x | X-ray structure of human ribonuclease inhibitor complexed with ribonuclease i | x-ray diffraction | 1.95 |
| 4jpk | Crystal structure of the germline-targeting hiv-1 gp120 engineered outer domain eod-gt6 in complex with a putative vrc01 germline precursor fab | x-ray diffraction | 2.40 |
| 4rs1 | Crystal structure of receptor-cytokine complex | x-ray diffraction | 2.68 |
| 1emv | Crystal structure of colicin e9 dnase domain with its cognate immunity protein im9 (1.7 angstroms) | x-ray diffraction | 1.70 |
| 1sbb | T-cell receptor beta chain complexed with superantigen seb | x-ray diffraction | 2.40 |
| 1mlc | Monoclonal antibody fab d44.1 raised against chicken egg- white lysozyme complexed with lysozyme | x-ray diffraction | 2.50 |
| 1ohz | Cohesin-dockerin complex from the cellulosome of clostridium thermocellum | x-ray diffraction | 2.20 |
| 2abz | Crystal structure of c19a/c43a mutant of leech carboxypeptidase inhibitor in complex with bovine carboxypeptidase a | x-ray diffraction | 2.16 |
| 4cvw | Structure of the barley limit dextrinase-limit dextrinase inhibitor complex | x-ray diffraction | 2.67 |
| 4y61 | Crystal structure of the complex between slitrk2 lrr1 and ptp delta ig1-fn1 | x-ray diffraction | 3.36 |
| 4g0n | Crystal structure of wt h-ras-gppnhp bound to the rbd of raf kinase | x-ray diffraction | 2.45 |
| 2qja | Crystal structure analysis of bmp-2 in complex with bmpr-ia variant b12 | x-ray diffraction | 2.60 |
| 1wqj | Structural basis for the regulation of insulin-like growth factors (igfs) by igf binding proteins (igfbps) | x-ray diffraction | 1.60 |
| 2qjb | Crystal structure analysis of bmp-2 in complex with bmpr-ia variant ia/ib | x-ray diffraction | 2.50 |
| 4krp | Nanobody/vhh domain 9g8 in complex with the extracellular region of egfr | x-ray diffraction | 2.82 |
| 3bp8 | Crystal structure of mlc/eiib complex | x-ray diffraction | 2.85 |

**Table S2** - Distribution of multiple point mutations across different experimental methods available in SKEMPI2 and used in this work.

| **Acronym** | **Technique** | **# mutations** |
| --- | --- | --- |
| SPR | Surface Plasmon Resonance | 599 |
| FL | Fluorescence | 355 |
| SFFL | Stopped Flow Fluorescence | 170 |
| ITC | Isothermal Titration | 138 |
| SP | Spectroscopy | 115 |
| IASP | Spectroscopy Inhibition Assay | 81 |
| ELISA | ELISA | 70 |
| RA | Radioactive Ligand Binding | 63 |
| IAFL | Fluorescence Inhibition Assay | 54 |
| KinExA | Kinetic Exclusion Assay | 47 |
| IARA | Radioligand Inhibition Assay | 11 |
| ELFA | Enzyme-linked Functional Assay | 10 |
| BI | Biolayer Interferometry | 3 |
| Other | SE, IAGE and ESMA | 3 |
| SPR,SFFL | SPR,SFFL | 2 |

**Table S3** - Complementary features used to model the effects of multiple point mutations on PPIs.

| **Category** | **Description** | **Tool** |
| --- | --- | --- |
| Normal Mode Analysis | Deformation energy and atomic fluctuation across 4 different force-fields (C-alpha, ANM, pfANM, REACH, sdENM) | Bio3D (1) |
| Residue Environment | Torsion angles (psi and phi), relative solvent accessibility and residue depth | Biopython (2) |
| Evolutionary and contact potential | Scores from substitution tables | AAINDEX (3), Blosum and PAM matrices |
| Non-covalent contacts | Hydrogen bonds, Hydrophobic contacts, PI stacking and Ionic interactions | Arpeggio (4) |
| Wild-type inter-residue distance | Average, shortest and longest distances among wild-type residues being mutated | Python |
| Individual ΔΔG^binding^ | Calculated for each single-point mutation separately | mCSM-PPI2 (5) |

**Table S4** - Performance of mmCSM-PPI for different supervised learning algorithms. Evaluation metrics were calculated using 10-fold cross validation before feature selection.

| **Algorithm** | **Pearson** | **Kendall** | **Spearman** | **RMSE (kcal/mol)** |
| --- | --- | --- | --- | --- |
| Extra Trees | 0.73 | 0.53 | 0.73 | 1.66 |
| Random Forest | 0.70 | 0.52 | 0.72 | 1.75 |
| Gradient Boosting | 0.69 | 0.50 | 0.70 | 1.79 |
| XGBoost | 0.68 | 0.49 | 0.68 | 1.81 |

**Table S5** - Parameters used in the final predictive model for mmCSM-PPI using Extra Trees algorithm available in the Scikit-learn Python library.

| **Hyperparameter** | **Values** |
| --- | --- |
| n_estimators | 300 |
| min_samples_split | 10 |
| min_samples_leaf | 3 |
| max_depth | 40 |
| bootstrap | False |

**Table S6** - Feature importance from the Extra Trees algorithm used for the final model of mmCSM-PPI.

| **Feature** | **Score** |
| --- | --- |
| Sum of ΔΔG^binding^ for each single point mutation separately (mCSM-PPI2) | 0.480 |
| Average graph-based signatures of wild-type residues | 0.462 |
| Sum of scores from DOSZ010103 (AAINDEX) | 0.015 |
| Average Pharmacophore changes (Positives) | 0.007 |
| Average deformation energies of wild-type residues (Bio3D) | 0.006 |
| Sum of Pharmacophore changes (Sulfurs) | 0.005 |
| Sum of scores from RUSR970103 (AAINDEX) | 0.004 |
| Sum of deformation energies of wild-type residues (Bio3D) | 0.004 |
| Sum of Hydrophobic contacts of wild-type residues (Arpeggio) | 0.003 |
| Average atomic fluctuation of wild-type residues (Bio3D) | 0.003 |
| Average Weak Polar interactions of wild-type residues (Arpeggio) | 0.003 |
| Sum of scores for MEHP950101 (AAINDEX) | 0.003 |
| Shortest distance between wild-type residues | 0.003 |
| Sum of Pharmacophore changes (Negatives) | 0.002 |
| Average Phi torsion angle of wild-type residues | 0.002 |

**Table S7** - Performance comparison of mmCSM-PPI and predictive models using only the most important features on a non-redundant blind test comprising entries with 4 or more mutations.

|  | Pearson | Kendall | Spearman | RMSE |
| --- | --- | --- | --- | --- |
| mmCSM-PPI | 0.70 | 0.48 | 0.64 | 2.06 |
| Sum of Individual ΔΔGbinding | 0.61* | 0.41# | 0.57+ | 2.55a |
| Graph-based signatures | 0.37* | 0.26# | 0.37+ | 2.83a |

* p-value < 0.05 by Fisher r-to-z transformation test
# p-value < 0.05 by transforming tau-to-r followed by Fisher r-to-z transformation

+ p-value < 0.05 by transforming rho-to-r followed by Fisher r-to-z transformation
a p-value < 0.05 by Diebold‐Mariano test

**Table S8**- Distribution of multiple mutations across different protein-protein complex structures in the PDB extracted from SKEMPI2.

| **Protein-protein complex (PDB)** | **# multiple mutations** |
| --- | --- |
| 1JTG | 136 |
| 3SGB | 88 |
| 1CHO | 84 |
| 1KBH | 83 |
| 1R0R | 76 |
| 2B2X | 67 |
| 3S9D | 61 |
| 1PPF, 1AO7 | 56 |
| 1BRS | 45 |
| 4G0N | 42 |
| 1A22 | 37 |
| 3L5X | 34 |
| 1KNE | 33 |
| 3SE3 | 32 |
| 2C5D, 1LFD | 31 |
| 1DAN | 29 |
| 1REW | 25 |
| 2VN5 | 24 |
| 3MZW | 22 |
| 1MHP | 20 |
| 2G2U, 5XCO | 18 |
| 2WPT | 17 |
| 4NKQ, 3HFM, 1MLC, 1CZ8 | 16 |
| 3KUD | 15 |
| 1HE8, 1VFB, 1BP3 | 14 |
| 1EMV, 1DQJ, 1A4Y, 1DVF | 13 |
| 3VR6, 4MNQ, 1JRH, 1BJ1 | 12 |
| 3IDX, 2NY7 | 11 |
| 1QAB, 4K71, 4FTV | 9 |
| 3HG1, 4RS1, 4UYP, 4UYQ, 5E9D | 8 |
| 4RA0, 1AHW, 1MQ8, 5M2O, 1BD2 | 7 |
| 3BE1, 3G6D, 2PCC, 2J0T, 4L3E | 6 |
| 2KSO, 2DVW, 2PYE, 4JFE, 4JFD, 1OHZ, 3BDY, 1YCS | 5 |
| 2OOB, 1FSS, 1Z7X, 1TM1, 2P5E, 1MAH, 3NGB | 4 |
| 2QJA, 5UFE, 1YY9, 4GNK, 2QJ9, 5CYK, 2QJB, 4YH7, 4E6K, 1GUA, 4J2L, 1C1Y, 3D3V, 4B0M | 3 |
| 3EG5, 1GC1, 1YQV, 2NYY, 4OFY, 1B41, 1N8Z, 3QIB, 5UFQ, 3QDG, 3QDJ, 4YFD, 3SE4, 5C6T | 2 |
| 2ABZ, 4KRL, 1FCC, 1E50, 1XXM, 3PWP, 1C4Z, 3BP8, 4JEU, 1EFN, 1AK4, 4MYW, 4CVW, 4X4M, 1WQJ, 4GXU, 2NOJ, 1B2S, 2NZ9, 3AAA, 2O3B, 1B2U, 4KRP, 4Y61, 4KRO, 4WND, 3UIH, 1GL0, 1GL1, 4G2V, 3LZF, 5TAR, 3U82, 3MZG, 1K8R, 1Y4A, 4JPK, 4GU0, 3UII, 3M63, 2CCL, 1B3S, 1SBB | 1 |

**Table S9** - Performance comparison on increasing and decreasing mutations.

|  | **Decreasing affinity** | | | | **Increasing affinity** | | | |
| --- | --- | --- | --- | --- | --- | --- | --- | --- |
| Method | Pearson | Kendall | Spearman | RMSE | Pearson | Kendall | Spearman | RMSE |
| mmCSM-PPI | 0.72 | 0.46 | 0.64 | 1.67 | 0.16 | 0.21 | 0.31 | 2.93 |
| Discovery Studio | 0.30^*^ | 0.30^#^ | 0.44^+^ | 4.84^a^ | 0.18 | 0.11 | 0.17 | 5.42 |
| FoldX | 0.34^*^ | 0.21^#^ | 0.32^+^ | 2.83^a^ | 0.23 | 0.20 | 0.32 | 2.83 |

^*^ p-value < 0.05 by Fisher r-to-z transformation test
^#^ p-value < 0.05 by transforming tau-to-r followed by Fisher r-to-z transformation

^+^ p-value < 0.05 by transforming rho-to-r followed by Fisher r-to-z transformation
^a^ p-value < 0.05 by Diebold‐Mariano test

**Table S10** - mmCSM-PPI classification by regression using different thresholds.

|  |  | **\|ΔΔG^binding^\| > 0.5** | | | | **\|ΔΔG^binding^\| > 1.0** | | | | **\|ΔΔG^binding^\| > 1.5** | | | |
| --- | --- | --- | --- | --- | --- | --- | --- | --- | --- | --- | --- | --- | --- |
| **Threshold** | **Class** | **Precision** | **Recall** | **MCC** | **AUC** | **Precision** | **Recall** | **MCC** | **AUC** | **Precision** | **Recall** | **MCC** | **AUC** |
| ΔΔG^mmCSM-PPI^ > -1.0 | Increase | 0.46 | 0.76 | 0.44 | 0.76 | 0.45 | 0.80 | 0.47 | 0.78 | 0.45 | 0.79 | 0.48 | 0.79 |
| ΔΔG^mmCSM-PPI^ < -1.0 | Decrease | 0.92 | 0.75 | 0.44 | 0.76 | 0.94 | 0.77 | 0.47 | 0.78 | 0.95 | 0.80 | 0.48 | 0.79 |
| ΔΔG^mmCSM-PPI^ > -0.50 | Increase | 0.52 | 0.61 | 0.43 | 0.73 | 0.53 | 0.65 | 0.48 | 0.76 | 0.52 | 0.65 | 0.48 | 0.76 |
| ΔΔG^mmCSM-PPI^ < -0.50 | Decrease | 0.88 | 0.84 | 0.43 | 0.73 | 0.91 | 0.86 | 0.48 | 0.76 | 0.92 | 0.87 | 0.48 | 0.76 |
| ΔΔG^mmCSM-PPI^ > 0 | Increase | 0.76 | 0.43 | 0.49 | 0.70 | **0.74** | **0.49** | **0.53** | **0.72** | 0.77 | 0.48 | 0.55 | 0.73 |
| ΔΔG^mmCSM-PPI^ < 0 | Decrease | 0.86 | 0.96 | 0.49 | 0.70 | **0.89** | **0.96** | **0.53** | **0.72** | 0.90 | 0.97 | 0.55 | 0.73 |
| ΔΔG^mmCSM-PPI^ > 0.50 | Increase | 0.94 | 0.29 | 0.46 | 0.64 | 0.94 | 0.36 | 0.53 | 0.68 | 0.96 | 0.39 | 0.57 | 0.69 |
| ΔΔG^mmCSM-PPI^ < 0.50 | Decrease | 0.83 | 0.99 | 0.46 | 0.64 | 0.87 | 0.99 | 0.53 | 0.68 | 0.8 | 1.00 | 0.57 | 0.69 |
| ΔΔG^mmCSM-PPI^ > 1.00 | Increase | 1.00 | 0.18 | 0.38 | 0.59 | 1.00 | 0.24 | 0.45 | 0.62 | 1.00 | 0.24 | 0.46 | 0.62 |
| ΔΔG^mmCSM-PPI^ < 1.00 | Decrease | 0.81 | 1.00 | 0.38 | 0.59 | 0.84 | 1.00 | 0.45 | 0.62 | 0.86 | 1.00 | 0.46 | 0.62 |

**Table S11** - Performance comparison for blind-test non-redundant at the mutation level.

| Method | Pearson | Kendall | Spearman | RMSE (kcal/mol) |
| --- | --- | --- | --- | --- |
| mmCSM-PPI | 0.67 | 0.47 | 0.67 | 1.72 |
| Discovery Studio | 0.36* | 0.28# | 0.38+ | 2.74a |
| FoldX | 0.29* | 0.26# | 0.39+ | 4.55a |

* p-value < 0.05 by Fisher r-to-z transformation test
# p-value < 0.05 by transforming tau-to-r followed by Fisher r-to-z transformation

+ p-value < 0.05 by transforming rho-to-r followed by Fisher r-to-z transformation
a p-value < 0.05 by Diebold‐Mariano test

# FIGURES


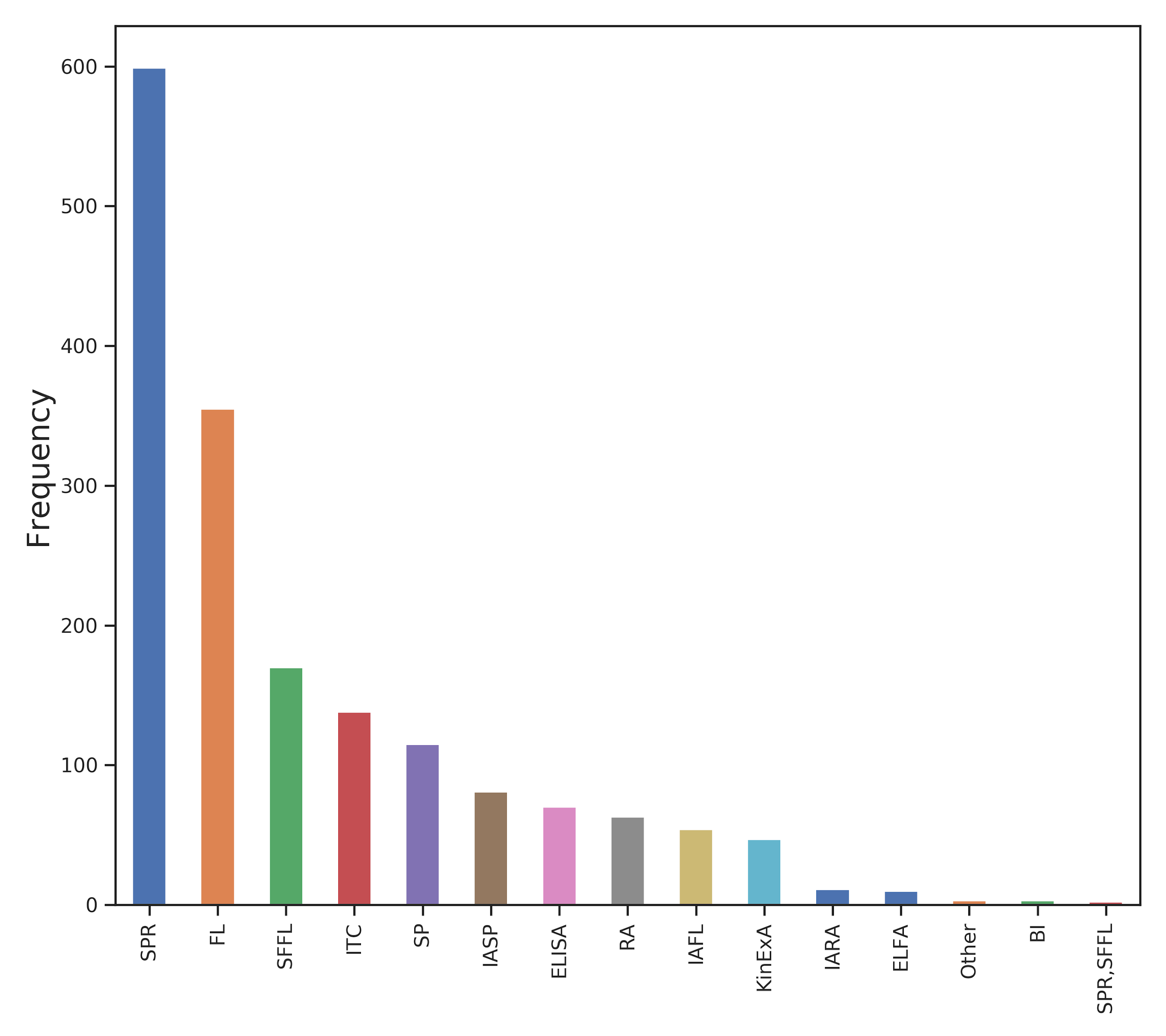


**Figure S1** - Distribution of experimental techniques used to generate the data set extracted from SKEMPI2. 5 experimental techniques represent the majority of the dataset (80%): surface plasmon resonance (SPR), fluorescence (FL), stopped flow fluorescence (SFFL), isothermal titration calorimetry (ITC) and spectroscopy.


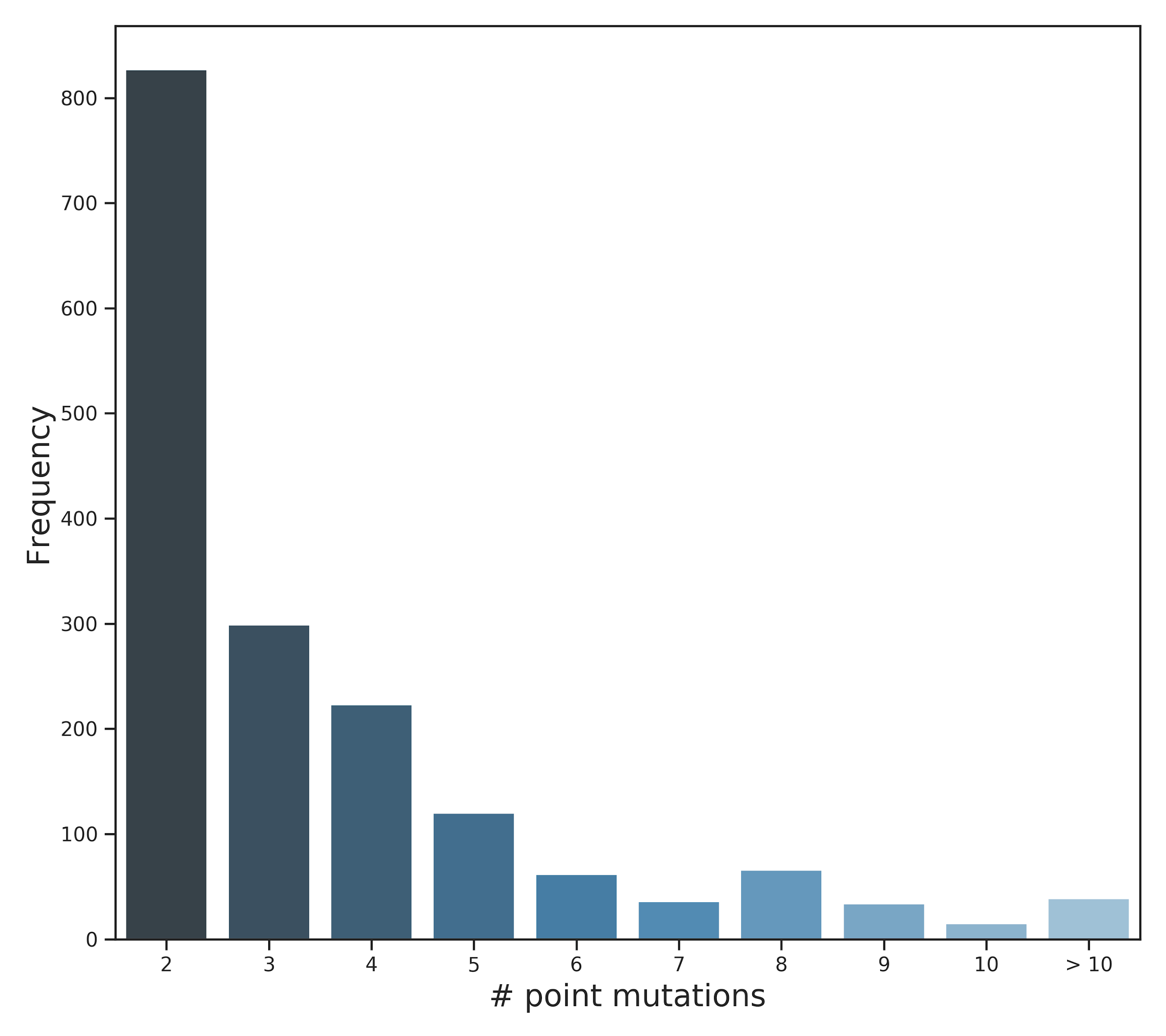


**Figure S2** - Distribution of multiple mutations across the data retrieved from SKEMPI2. Double and triple mutants, which account for more than 65% (1126) of all entries, were used for training mmCSM-PPI. The remaining 595 entries were held out and used as a non-redundant blind test at mutation level.


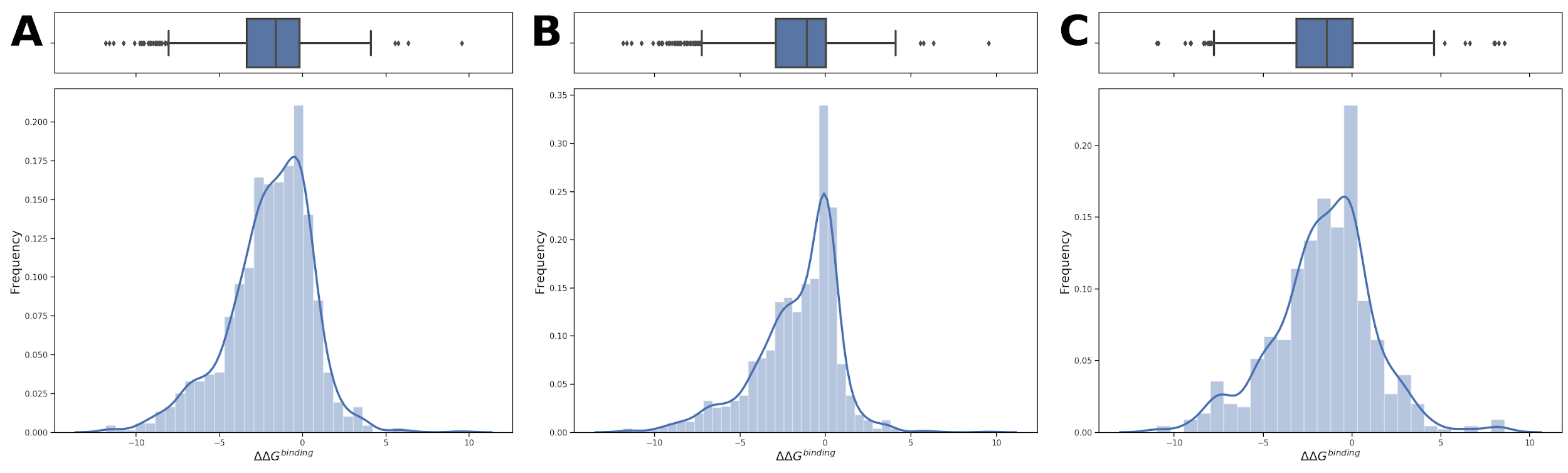


**Figure S3** - ΔΔG^binding^ distribution of training and blind test sets used in this work. A) depicts the distribution of changes in binding affinity for the original training set extracted from SKEMPI2. B) shows the distribution on the training set after including hypothetical reverse mutations. Finally, C) summarises the ΔΔG^binding^ on the non-redundant blind test set used for validation and comparisons with other methods.


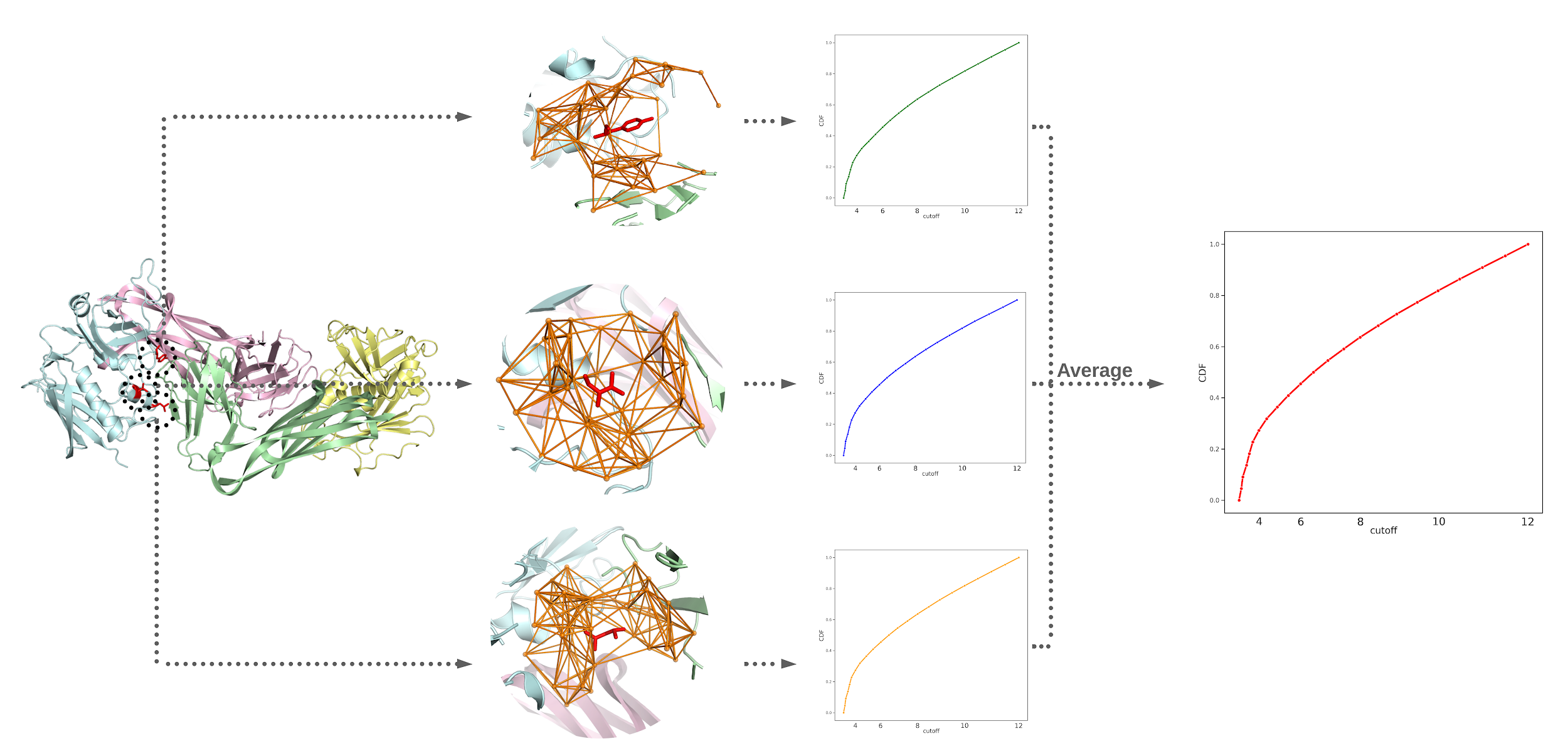


**Figure S4** - Graph-based signatures representation for multiple point mutations. For each point mutation, the residue environment is represented as a graph where surrounding residues are represented as nodes and their interactions as edges. Distance patterns between atoms characterised by their properties are compiled as cumulative distributions for each environment separately. Finally, the distributions are averaged based on the number of point mutations.


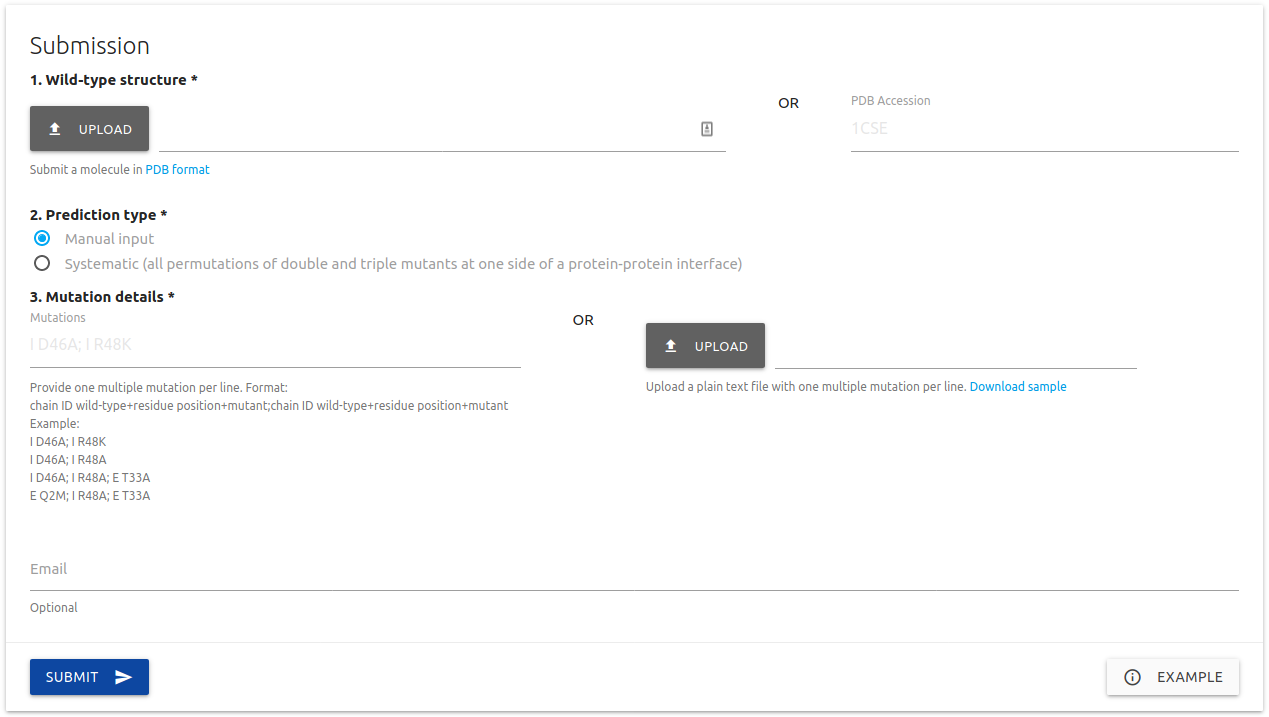


**Figure S5** - mmCSM-PPI submission page. Two predictive types are available: Manual input and Systematic evaluation. In both cases, users must provide the 3D structure of a protein complex by either uploading a file in PDB format or specifying a valid PDB accession code. For the Manual input option, a list of mutations of interest are required, represented as the chain identifier, the wild-type residue one-letter code, the residue number and the mutant residue one-letter code, and with each point mutation separated by a semi-colon. For the Systematic evaluation option, users must specify a chain identifier from which interfaces are automatically identified and the effects of all double and triple point mutations are assessed.


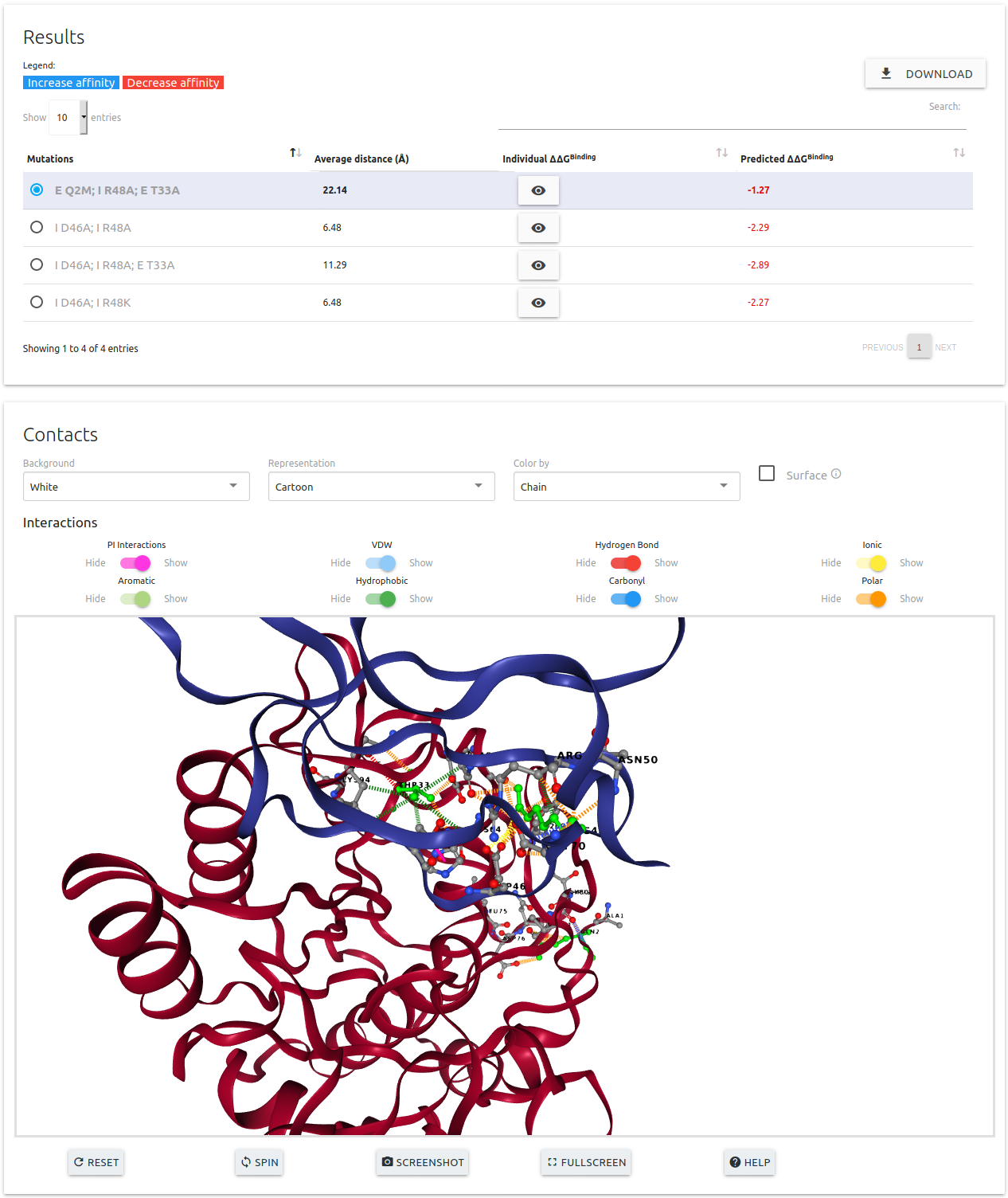


**Figure S6** - mmCSM-PPI results page. For both predictive options, manual input and systematic evaluation, results are shown as a downloadable table where predicted effects of multiple mutations in ΔΔGbinding and individual predicted effects for each single-point mutation are also available. In addition, an interactive 3D viewer is also available where interactions for an entry are displayed. Users can change the interactions in the interactive viewer by selecting entries from the table.

# REFERENCES

1. Grant, B.J., Rodrigues, A.P., ElSawy, K.M., McCammon, J.A. and Caves, L.S. (2006) Bio3d: an R package for the comparative analysis of protein structures. *Bioinformatics*, **22**, 2695-2696. <http://dx.doi.org/10.1093/bioinformatics/btl461>

2. Cock, P.J., Antao, T., Chang, J.T., Chapman, B.A., Cox, C.J., Dalke, A., Friedberg, I., Hamelryck, T., Kauff, F., Wilczynski, B. *et al.* (2009) Biopython: freely available Python tools for computational molecular biology and bioinformatics. *Bioinformatics*, **25**, 1422-1423. <http://dx.doi.org/10.1093/bioinformatics/btp163>

3. Kawashima, S. and Kanehisa, M. (2000) AAindex: amino acid index database. *Nucleic Acids Res*, **28**, 374. <http://dx.doi.org/10.1093/nar/28.1.374>

4. Jubb, H.C., Higueruelo, A.P., Ochoa-Montano, B., Pitt, W.R., Ascher, D.B. and Blundell, T.L. (2017) Arpeggio: A Web Server for Calculating and Visualising Interatomic Interactions in Protein Structures. *J Mol Biol*, **429**, 365-371. <http://dx.doi.org/10.1016/j.jmb.2016.12.004>

5. Rodrigues, C.H.M., Myung, Y., Pires, D.E.V. and Ascher, D.B. (2019) mCSM-PPI2: predicting the effects of mutations on protein-protein interactions. *Nucleic Acids Res*, **47**, W338-W344. <http://dx.doi.org/10.1093/nar/gkz383>
